# Structural determination of the lipid A from the deep-sea bacterium *Zunongwangia profunda* SM-A87: a small-scale approach

**DOI:** 10.1007/s10719-022-10076-6

**Published:** 2022-08-05

**Authors:** Molly Dorothy Pither, Mei-Ling Sun, Immacolata Speciale, Alba Silipo, Yu-Zhong Zhang, Antonio Molinaro, Flaviana Di Lorenzo

**Affiliations:** 1 Department of Chemical Sciences, University of Naples Federico II, via Cinthia, 80126 Naples, Italy; 2 College of Marine Life Sciences and Frontiers Science Center for Deep Ocean Multispheres and Earth System, Ocean University of China, Qingdao, People’s Republic of China; 3 Laboratory for Marine Biology and Biotechnology, Pilot National Laboratory for Marine Science and Technology, Qingdao, People’s Republic of China; 4 Department of Agricultural Sciences, University of Naples Federico II, Via Università, 80055 Portici, Naples, Italy; 5 State Key Laboratory of Microbial Technology, Marine Biotechnology Research Center, Shandong University, Qingdao, People’s Republic of China

**Keywords:** Deep-sea bacteria, *Zunongwangia*, Lipopolysaccharide, Lipid A, Structural characterization, MALDI-TOF MS

## Abstract

*Zunongwangia profunda* SM-A87 is a deep-sea sedimentary bacterium from the phylum Bacteroidetes, representing a new genus of *Flavobacteriaceae*. It was previously investigated for its capability of yielding high quantities of capsular polysaccharides (CPS) with interesting rheological properties, including high viscosity and tolerance to high salinities and temperatures. However, as a Gram-negative, *Z. profunda* SM-A87 also expresses lipopolysaccharides (LPS) as the main components of the external leaflet of its outer membrane. Here, we describe the isolation and characterization of the glycolipid part of this LPS, i.e. the lipid A, which was achieved by-passing the extraction procedure of the full LPS and by working on the ethanol precipitation product, which contained both the CPS fraction and bacterial cells. To this aim a dual approach was adopted and all analyses confirmed the isolation of *Z. profunda* SM-A87 lipid A that turned out to be a blend of species with high levels of heterogeneity both in the acylation and phosphorylation pattern, as well as in the hydrophilic backbone composition. *Mono*-phosphorylated tetra- and penta-acylated lipid A species were identified and characterized by a high content of branched, odd-numbered, and unsaturated fatty acid chains as well as, for some species, by the presence of a hybrid disaccharide backbone.

## Introduction

The deep-sea is a complex and mysterious ecosystem breeding an enormous variety of life forms, which includes the largest fraction of bacterial taxonomic richness and biomass on Earth [1, 2]. This phenomenon is extremely intriguing as deep-sea environments, with their water depth more than 1000 m, typically exhibit harsh abiotic conditions encompassing high hydrostatic pressure, low temperatures, high salinity, absence of sunlight, and a low input of organic matter [1, 2]. Therefore, deep-sea bacteria must employ a variety of adaptive strategies to survive and proliferate in a habitat that is lethal to other microorganisms. In the large portfolio of adopted strategies, the production of the so-called “extremozymes” is certainly the most studied. These enzymes exhibit great thermal or cold adaptability, salt and/or pressure tolerance, opening to numerous applications in a wide range of industries, including the chemical, pharmaceutical, and biotechnological sectors [3]. In addition, a plethora of deep-sea bacteria, in response to a stress, are also able to produce large amounts of extracellular polysaccharides that serve disparate functions, such as the formation of a microenvironment that facilitates attachment to biotic or abiotic surfaces, capture of nutrients, and protection against toxins and other stressors of the surroundings [4]. One of these extracellular polysaccharide-producing deep-sea bacteria is *Zunongwangia profunda* SM-A87. This is a Gram-negative bacterium isolated from subseafloor sediments of the southern Okinawa Trough, a seabed feature of the East China Sea, where the water depth reaches 1245 m and the *in-situ* temperature is 4.7 °C [5]. The highly viscous polysaccharide produced by *Z. profunda* SM-A87 has been the focus of various research activities, showing a potential for biotechnological and biomedical uses including the capacity to enhance oil recovery, to absorb Cu(II) and Cd(II) [6, 7], and to display anti-oxidant properties in scavenging free radicals [8]. Of note, this massive secretion of polysaccharide(s) results in the formation of a thick capsule (CPS) connected to the bacterial outer membrane that protects cells and allows to concentrate proteinaceous particles and metal ions as a growth and energy source [9].

It is worth to underline that, as a Gram-negative, *Z. profunda* SM-A87 is externally covered by an additional layer of another kind of glycoconjugate, i.e. lipopolysaccharides (LPS), which instead represent the main constituents of the outer membrane. LPS are amphiphilic macromolecules with a tripartite structural architecture that typically comprises: a glycolipid part (the lipid A) embedded in the outer membrane, which is connected to an oligosaccharide (the core OS), in turn linked to a highly variable polysaccharide chain (the O-antigen) [10]. An LPS can be classified as smooth or rough depending on the nature of the carbohydrate portion: smooth-type LPS (S-LPS) is built up of all the three above domains, whereas a rough-type LPS (R-LPS) does not express any O-antigen moiety [10]. Within each of the three individual components of an LPS, structural discrepancies have been identified across bacterial species and within the same species [10]. Most of these differences have been associated to mechanisms by which bacteria adapt to the surrounding environment [11].Indeed, it is largely known that bacteria can colonize an inhospitable habitat by modifying the chemical structure of their LPS to reinforce the cell envelope thus favouring viability and survival [11]. As a direct consequence of this adaptation phenomenon, LPS of bacteria inhabiting extreme environments, such as deep-sea, are characterized by several uncommon structural features, which render these microorganisms extraordinarily fascinating under both an evolutionary and chemical point of view. On the other hand, LPS are also widely acknowledged to be the main bacterial molecules interacting with the host immune system, with the lipid A moiety specifically recognized by the innate immunity receptorial complex Toll-Like Receptor 4/myeloid differentiation factor-2 (TLR4/MD-2) [10]. As a result of this interaction, pathogenic bacteria can induce an inflammatory reaction leading, in certain cases, to fatal outcomes for human health [12]. The activation of the TLR4/MD-2-dependent response is, however, highly dependent on the chemical structure of the lipid A. This implies that on the basis of the lipid A structure, an LPS is able to differently activate the production of host pro-inflammatory mediators. In fact, the reported immunological properties of LPS/lipid A span from potent activation (commonly referred to as agonism) to only weak or no immunostimulatory action [10, 11]. By contrast, LPS/lipid A structures that are ineffective in activating the TLR4-mediated signalling while keeping the capability to bind the receptor and to prevent the binding of agonistic LPS are defined as antagonists [10, 11]. In this frame, the search for novel lipid A structures [10–15] that might possess any immunomodulatory activity towards TLR4/MD-2-dependent signalling is of high relevance for researchers of both the biomedical and pharmacological field who are attracted by the possibility to finely tune the immune response and, hence the inflammatory process(es). Consequently, due to the intrinsic nature of living and thriving in extreme habitats, deep-sea bacteria are evaluated as harmless for humans, therefore they can be considered as a “gold mine” for the discovery of new LPS and lipid A molecules to be isolated and analysed for the realization of new generation immune-therapeutics inspired by a natural source.

Within this frame, here we report the characterization of the chemical structure of the lipid A isolated from *Z. profunda* SM-A87, obtained by combining data attained from different approaches including Gas Chromatography-Mass Spectrometry (GC–MS), Matrix-Assisted Laser Desorption Ionization-Time Of Flight (MALDI-TOF) MS, and tandem MS (MS/MS). Two parallel approaches were adopted starting from the cold ethanol precipitation product (CPS-EtOH), which encompassed both the CPS and traces of bacterial cells. These approaches allowed to identify for *Z. profunda* SM-A87 a heterogenous blend of *mono*-phosphorylated tetra- and penta-acylated lipid A species characterized by a high content of branched, odd-numbered, and unsaturated acyl chains as well as by the presence of a hybrid disaccharide backbone. Of note, this structural deduction was then definitively confirmed by the MALDI-TOF MS and MS/MS analysis performed directly on an aliquot of lyophilized cellular pellet.

## Materials and methods

### Isolation and purification of the R-LPS and lipid A fraction

*Z. profunda* SM-A87 (CCTCC AB 206139^ T^ = DSM 18,752) was inoculated into a liquid marine medium (pH 8.5) containing 10 g/L peptone, 5 g/L yeast extract and 30 g/L sea salt, and then incubated at 15 °C, with gentle shaken (200 rpm) for 60 h to logarithmic phase. Following, a 2% (vol/vol) inoculum of this culture was added to the fermentation medium (pH 8.5) containing 32.21 g/L lactose, 8.87 g/L peptone, 7.5 g/L yeast extract and 30 g/L sea salt [8]. After incubation at 9.8 °C, 200 rpm for 6 days (atmospheric pressure), an aliquot of the fermentation broth was mixed with 2 volumes of cold absolute ethanol to precipitate the CPS and centrifuged for 10 min at 10,000 rpm. In addition, to remove low molecular weight impurities, the precipitation was washed twice by cold absolute ethanol. The resulting precipitate (CPS-EtOH), which contained both the CPS and bacterial cells, was suspended in distilled water and lyophilized. In order to allow the separation of cells and the CPS, an aliquot of dried CPS-EtOH (30 mg) was ultracentrifuged (208,000 × *g*, 4 °C, for 16 h). Both the supernatant (containing the CPS) and the precipitate (containing bacterial cells) of the ultracentrifugation were collected separately, lyophilized, and checked via Sodium Dodecyl Sulphate—PolyAcrylamide Gel Electrophoresis (SDS-PAGE) upon a proper enzymatic digestion. Briefly, an aliquot (1 mg) of both dried samples were diluted using 1 mL of a prepared buffer solution [0.5 M Tris HCl (pH 6.8), glycerol (20% vol/vol), mercaptoethanol (4% vol/vol), SDS (4% wt/vol), and bromophenol blue (0.2% wt/vol)] and boiled for 10 min. Following, 10 µL of proteinase K (5 mg/mL) (Merck) was added and samples were digested at 56 °C for 1 h. Samples were then diluted with water-saturated phenol and vortexed for a few seconds, then incubated at 68 °C for 30 min. 1 mL of diethyl ether was added to each sample, vortexed, and centrifuged (8,800 × *g*, 15 min). Collected the bottom layers, these were then diluted 1:10 with sample buffer [16]. 10 μL of so-obtained digested samples were loaded on a 13.5% SDS-PAGE gel prepared with a 5% stacking gel and separated using a mini-PROTEAN electrophoresis instrument (Bio-Rad Laboratories).

LPS was found only in the precipitate (UCF_prec_) of the ultracentrifugation step. Therefore, to isolate the lipid A moiety, an aliquot of UCF_prec_ (~ 1 mg) was washed with 500 μL of chloroform–methanol (1:2, vol/vol) and then with 500 μL of chloroform–methanol-water (3:2:0.25, vol/vol/vol) followed by several steps of centrifugation (1,200 × *g*, 30 min). At this stage, UCF_prec_ underwent a slightly modified El Hamidi *et al*. [17] procedure to (micro)extract the lipid A from cells (**LipA**_**me**_). Briefly, pre-washed UCF_prec_ was suspended in 500 μL of isobutyric acid acid/1 M ammonium hydroxide (5:3 vol/vol) and left at 100 °C for 1 h. Upon centrifugation (1,200 × *g*, 30 min) the supernatant was collected, lyophilized, and washed several times with methanol, followed by a wash with chloroform–methanol-water (3:1.5:0.25, vol/vol). Then, the sample was prepared for chemical and MALDI-TOF MS analyses as described below. In parallel, another aliquot of cells was directly analyzed by MALDI-TOF MS following Larrouy-Maumus *et al*. method [18].

Finally, an aliquot of CPS-EtOH (30 mg) was suspended in 1% vol. acetic acid and hydrolyzed at 100 °C for 2 h. Methanol and chloroform were added to the reaction mixture to reach a CH_3_OH/CHCl_3_/hydrolysate 2:2:1.8 (v/v/v) ratio. This mixture was then shaken and centrifuged (4 °C, 8,800 × *g*, 30 min). Then, the chloroform phase was collected and transferred in a clean tube where it was washed with CH_3_OH and water to reach again a CHCl_3_/CH_3_OH/H_2_O, 2:2:1.8 ratio [19]. This washing step was executed three times. Then, the organic phases, containing the lipid A fraction (**LipA**_**ah**_) were pooled, dried, and analyzed as described below.

### Compositional analyses of lipid A fraction(s)

To evaluate the nature of the fatty acids and monosaccharides composing the lipid A of *Z. profunda* SM-A87, we proceeded by analysing both **LipA**_**me**_ and **LipA**_**ah**_. An aliquot of each sample underwent methanolysis (1.25 M HCl in CH_3_OH, 85 °C, 16 h) followed by acetylation (80 °C, 20 min) and GC–MS analysis [20, 21]. In addition, an aliquot of both **LipA**_**me**_ and **LipA**_**ah**_ was also hydrolyzed using 2 M TFA solution at 120 °C for 4 h. Then, reduction with NaBH_4_ and acetylation twice with equal amounts of acetic anhydride in pyridine (85 °C, 15 min) were performed [20]. The so-obtained acetylated alditols were analyzed by GC–MS.

As for the fatty acid content, this was ascertained by treating **LipA**_**me**_ and **LipA**_**ah**_ with 4 M HCl (100 °C, 4 h), followed by a treatment with 5 M NaOH (100 °C, 30 min). Fatty acids were extracted in chloroform, after the adjustment of the pH (pH ~ 3), and then methylated with diazomethane, and inspected by GC–MS. *O*-linked fatty acids were also analyzed by GC–MS after their release upon a treatment with aqueous 0.5 M NaOH in CH_3_OH (1:1, vol/vol, 85 °C, 2 h), followed by acidification of the products, extraction in chloroform and methylation with diazomethane. The absolute configuration of the 3-hydroxy fatty acids was defined after their release through a treatment with 4 M NaOH (100 °C, 4 h) and conversion into 3-methoxy acid L-phenylethylamides. These were then injected and analyzed via GC–MS [22]. The retention times of authentic L-phenylethylamides of standard fatty acids were compared with those derived from the examined lipid A products.

All the compositional analyses were executed on Agilent Technologies Gas Chromatograph 7820A equipped with a mass selective detector 5977B and an HP-5 capillary column (Agilent, Milan, Italy 30 m × 0.25 mm i.d., flow rate 1 mL/min, He as carrier gas). The temperature program was 140 °C for 5 min, 140 °C → 280 °C at 10 °C/min, and 280 °C for 10 min.

### MALDI-TOF MS and MS/MS analysis

MALDI-TOF MS and the MS/MS spectra were recorded in reflectron mode, both positive and negative-ion polarity mode on an ABSCIEX TOF/TOF 5800 Applied Biosystems (Foster City, CA, USA) mass spectrometer equipped with an Nd:YAG laser (*λ* = 349 nm), with a 3 ns pulse width and a repetition rate of up to 1000 Hz, and also equipped with delayed extraction technology. **LipA**_**ah**_ was dissolved in CHCl_3_/CH_3_OH (50:50, vol/vol) and the matrix solution used was 2,4,6-trihydroxyacetophenone (THAP) dissolved in CH_3_OH/0.1% trifluoroacetic acid/acetonitrile (7:2:1, vol/vol/vol) at a concentration of 75 mg/mL. This matrix was also used for **LipA**_**me**_ in place of 2,5-dihydroxybenzoic acid (DHB) in 0.1 M citric acid employed in the small-scale extraction protocol of El Hamidi *et al.* [17]. The use of THAP, in fact, provided better results for this analysis. To analyze bacterial cells (UCF_prec_), the matrix used was DHB (10 mg/mL) in chloroform/methanol (9:1, vol/vol) [18]. In all cases, 0.5 μL of the sample and 0.5 μL of the matrix solution were deposited onto a stainless-steel plate and left to dry at room temperature. For MS experiments each spectrum was a result of the accumulation of 2000 laser shots, whereas 5000–7000 shots were summed for the MS/MS spectra. Each experiment was performed in triplicate.

## Results

### Isolation and compositional analyses of the Lipid A fraction(s) from *Z. profunda* SM-A87 R-LPS

**Scheme 1:**
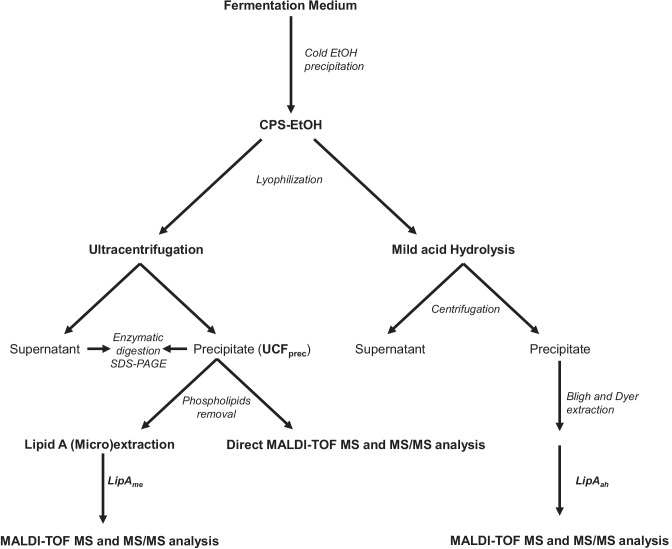
Schematic view of the methodological approach adopted to liberate, identify, and characterize the lipid A of *Z. profunda* SM-A8*7* starting from the ethanol-precipitate (CPS-EtOH) previously obtained to isolate the capsular polysaccharide produced by this deep-sea Gram-negative bacterium

**Fig. 1:**
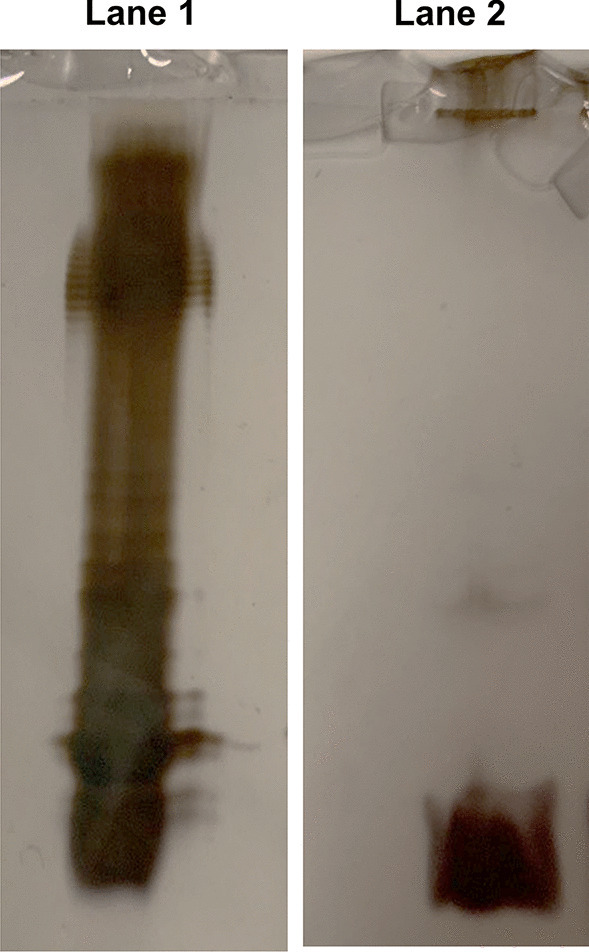
Sodium dodecyl sulphate–polyacrylamide gel electrophoresis (SDS-PAGE) after silver staining of *Z. profunda* SM-A8*7* R-LPS obtained upon enzymatic digestion of **UCF**_**prec**_ (Lane 2). S-LPS from *Escherichia coli* (Lane 1) was used as a benchmark

**Fig. 2:**
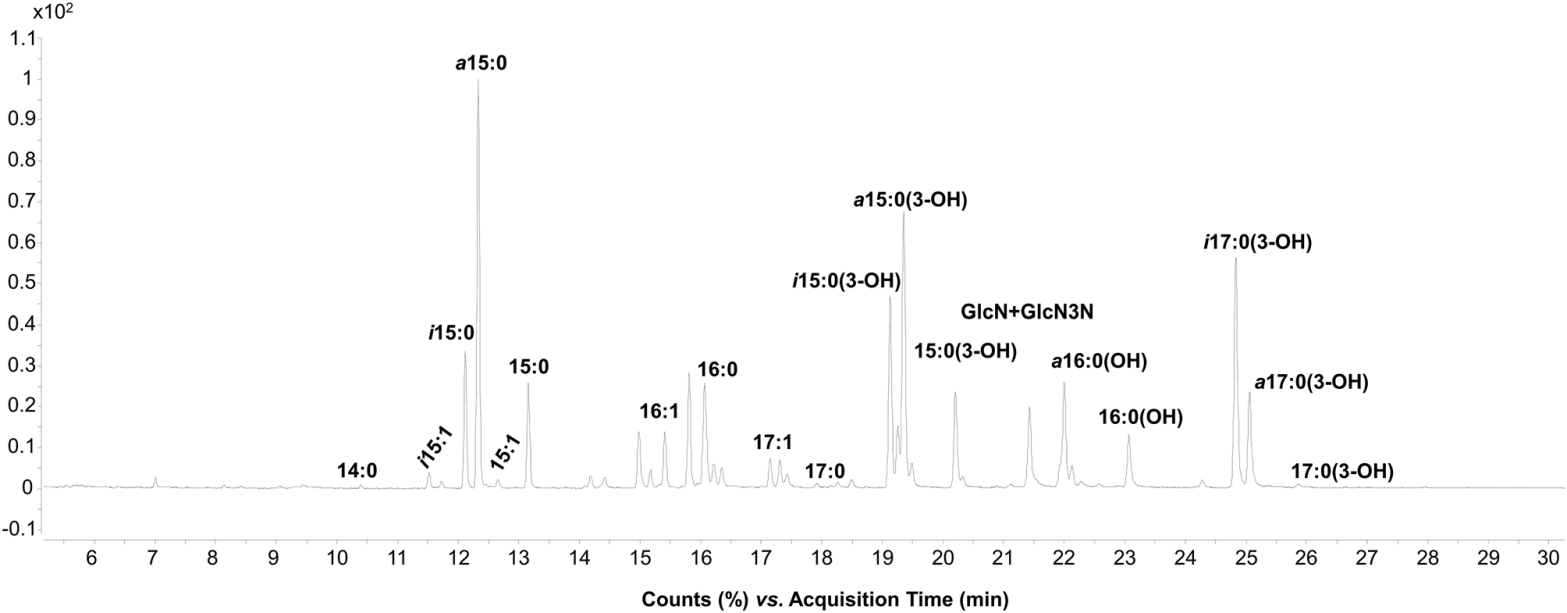
GC–MS chromatogram profile recorded after methanolysis followed by acetylation of an aliquot of the lipid A fraction isolated by (micro)extraction procedure (**LipA**_**me**_). By this approach fatty acids are detected as methyl esters whereas monosaccharides composing the lipid A sugar backbone as acetylated methyl glycosides

**Fig. 3:**
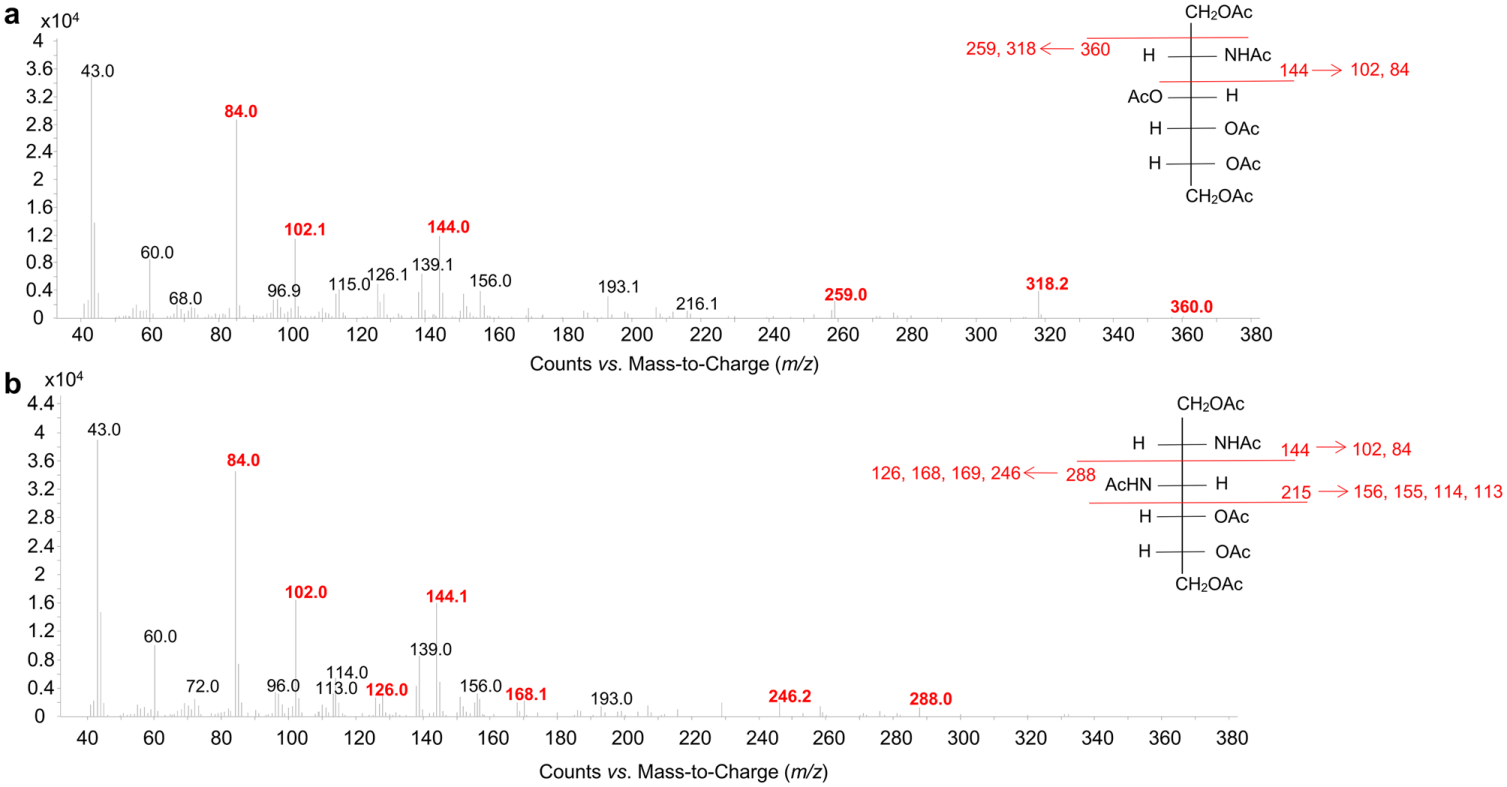
EI-mass spectra of the alditol acetate derivative of GlcN (**a**) and GlcN3N (**b**) found in *Z. profunda* SM-A8*7* lipid A. On the right, the fragmentation pattern observable for both residues is reported. Several daughter ions caused by elimination of acetic acid (− 60) or an *N*-acetyl group (− 42) from C3-C6 and C1-C2 fragment ions were observed, alongside with typical ions at *m/z* 156, 155, 114, and 113 that were matched with daughter ions generated from the C1-C3 fragment

To isolate and define the structure of the lipid A from *Z. profunda* SM-A87 LPS, two approaches were applied (Scheme 1). On the one hand, an aliquot of CPS-EtOH, containing both CPS and traces of bacterial cells, underwent a mild acid hydrolysis with aqueous acetic acid 1% followed by centrifugation to attempt the isolation of the lipid A as the water-insoluble fraction (**LipA**_**ah**_) of the centrifugation step. On the other hand, CPS-EtOH was ultracentrifuged, to allow the separation of bacterial cells (in the precipitate) and the CPS (in the supernatant). Both the supernatant and the precipitate were then lyophilized, enzymatically digested, and checked via SDS-PAGE after silver nitrate gel staining. This analysis highlighted the presence of LPS material only in the precipitate of the ultracentrifugation (UCF_prec_). Furthermore, it showed the “rough” nature of the *Z. profunda* SM-A87 LPS, as proven by the band migrating to the bottom of the gel, which is typical of a low molecular mass R-LPS, i.e. an LPS devoid of the O-antigen portion (Fig. 1). After removal of cellular phospholipids by using sequential washes with chloroform–methanol and chloroform–methanol-water mixtures, an aliquot of bacterial cells was (micro)extracted to isolate the lipid A fraction (**LipA**_**me**_). Then, both **LipA**_**me**_ and **LipA**_**ah**_ underwent a set of compositional analyses to establish the fatty acid and monosaccharide content. In particular, an aliquot of **LipA**_**me**_ and **LipA**_**ah**_ was subjected to methanolysis followed by an acetylation step in order to inspect the fatty acids as methyl ester derivatives and to define the nature of the lipid A saccharide backbone (Fig. 2). This analysis highlighted a heterogenous composition in terms of fatty acids present, which was however qualitatively equal for both **LipA**_**me**_ and **LipA**_**ah**_ (Table 1), and showed the occurrence, as expected, of the acetylated methyl glycoside derivative of 2-amino-2-deoxy-D-glucose (D-glucosamine). Of note, in both lipid A products, the analysis of the alditol acetate derivatives was key to also highlight the presence, in minor amount, of 2,3-diamino-2,3-dideoxy-D-glucose (GlcN3N). This was confirmed by the inspection of the electron impact (EI)-mass spectrum that showed characteristic fragment ions originated from the cleavage between C-2 and C-3 and detected at *m/z* 144 (C1 to C2 fragment) and *m/z* 288 (C3-C6 fragment), as shown in Fig. 3. This information, together with the total fatty acid analysis, was crucial in supporting the elucidation of the lipid A structure deduced by MALDI-TOF MS and MS/MS studies on **LipA**_**me**_ and **LipA**_**ah**_.

**Table 1:** Fatty acid content of **LipA**_**me**_ and **LipA**_**ah**_ isolated from *Z. profunda* SM-A87. Both lipid A fractions displayed the same fatty acid composition. All the 3-hydroxy fatty acids displayed an (*R*) configuration. For the unsaturated acyl chains the position of the double bond or the stereochemistry remain to be defined. Likewise, for *anteiso*-branched acyl chains the stereochemistry is tentatively given as (*S*) since it has been previously suggested to be predominant in bacteria, however it remains to be clarified

**Fatty Acid Component**
14:0
*iso* 15:1 (*i*15:1)
*i*15:0
*anteiso* 15:0 (*a*15:0)
15:1
15:0
16:1
16:0
*i*15:0(3-OH)
*a*15:0(3-OH)
15:0(3-OH)
*a*16:0(3-OH)
16:0(3-OH)
17:1
17:0
*i*17:0(3-OH)
*a*17:0(3-OH)
17:0(3-OH)

### MALDI-TOF MS and MS/MS investigation of the lipid A isolated from *Z. profunda* SM-A87

**Fig. 4:**
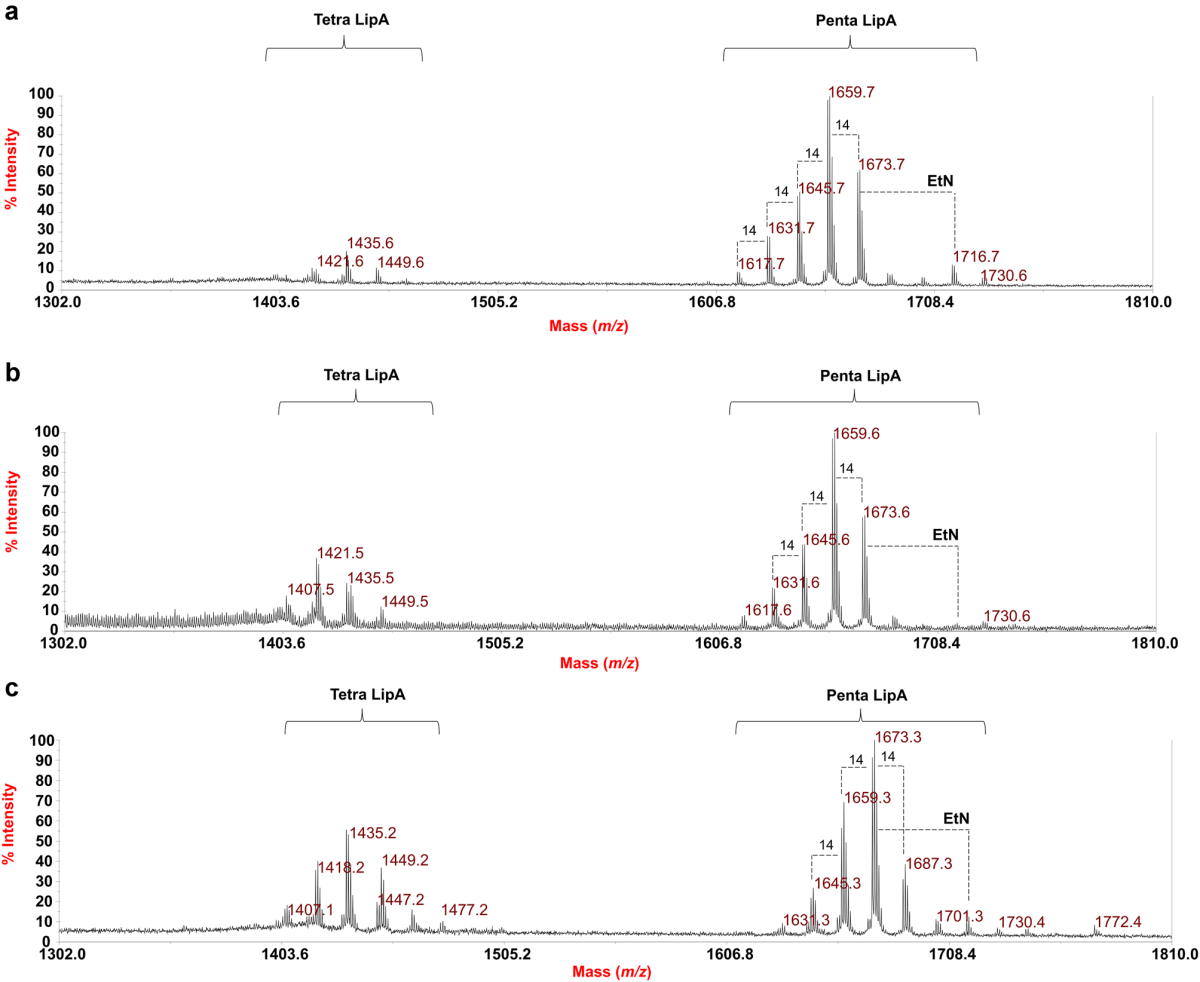
Reflectron MALDI-TOF MS spectra, recorded in negative-ion polarity, of **LipA**_**me**_ (**a**), **LipA**_**ah**_ (**b**), and bacterial pellet (**UCF**_**prec**_) (**c**). Lipid A species are labelled as Tetra LipA and Penta LipA on the basis of the degree of acylation. Differences of 14 amu, diagnostic for a methylene group, are also reported in the spectra.“**EtN**” indicates the 2-aminoethyl decoration on the *mono*-phosphorylated disaccharide backbone of the lipid A

Reflectron MALDI-TOF mass spectra, recorded in negative-ion polarity, of both **LipA**_**me**_ and **LipA**_**ah**_ are reported in Fig. 4a, b. MALDI-TOF MS spectra were almost identical, indicating the success of the approaches that were adopted to isolate the lipid A directly from CPS-EtOH. In addition, to complete the analysis, an aliquot of lyophilized cellular pellet (UCF_prec_) also underwent a direct MALDI-TOF MS analysis (Fig. 4c). This approach was necessary to confirm the structures deduced by studying the isolated lipid A fractions (**LipA**_**me**_ and **LipA**_**ah**_), and to exclude any loss of structural information possibly occurring due to the chemical treatments adopted to isolate the lipid A (i.e., the mild acid hydrolysis and the micro-extraction procedures). Hence, further corroborating our approach and structural deduction also the MALDI-TOF MS spectrum recorded on UCF_prec_ (Fig. 4c) was similar to those recorded for **LipA**_**me**_ and **LipA**_**ah**_ (Fig. 4a, b).

In all MS spectra (Fig. 4) two main families of peaks were identified and matched with deprotonated [M-H]^−^ *mono*-phosphorylated tetra-acylated (at about *m/z* 1435) and penta-acylated lipid A species (at about *m/z* 1659). The heterogeneity of *Z. profunda* SM-A87 lipid A was immediately evident as proven by the occurrence of peaks differing in 14 amu (–CH_2_– unit), which is indicative of the presence of lipid A species that differ in the length of their acyl chains. Further spectral complexity arose from lipid A structures bearing also unsaturated fatty acids (peaks differing in 2 amu), as well as lipid A species that possess GlcN3N instead of one GlcN in the disaccharide backbone, as also observed in compositional analyses. Moreover, the MALDI-TOF MS spectra showed the presence of an additional cluster of peaks at *m/z* 1716.7, *m/z* 1716.6, and *m/z* 1716.4 (Figs. 4a-c respectively) that was matched with penta-acylated lipid A species having a 2-aminoethyl substitution on the phosphate group. The occurrence of this family of lipid A species was more evident in the spectra recorded for **LipA**_**me**_ and UCF_prec,_ which suggested that the two protocols used (i.e. the microextraction and the direct analysis of the cellular pellet) were slightly milder than the one that entailed the use of acetic acid, thus also enabling the detection of labile substituents.

**Fig. 5:**
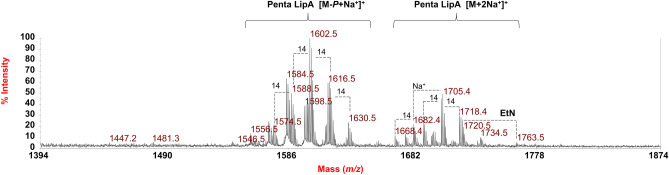
Zoom of the reflectron MALDI-TOF MS spectrum, recorded in positive-ion polarity, of **LipA**_**me**_. Lipid A species have been identified as both [M-*P* + Na^+^]^+^ and [M + 2Na^+^]^+^ species. Differences of 14 amu have been indicated in the spectrum. “**EtN**” indicates the 2-aminoethyl decoration on the *mono*-phosphorylated disaccharide backbone of the lipid A

Choosing as an example the spectrum recorded for **LipA**_**me**_, in the mass region *m/z* 1617.7–1701.7 (Fig. 4a) the main peak at *m/z* 1659.7 was attributed to a penta-acylated lipid A species built up of the typical glucosamine disaccharide backbone carrying one phosphate and bearing two 17:0(3-OH) [or *i*17:0(3-OH) or *a*17:0(3-OH)], two 15:0(3-OH) [or *i*15:0(3-OH) or *a*15:0(3-OH)], and one 15:0 [or *i*15:0 or *a*15:0] (Table 2). For description purposes of the MALDI-TOF MS and MS/MS characterization, hereafter the acyl moieties will be mentioned without indicating whether they are branched (and how) or not, taking into consideration that they can be also found in their *iso*- or *anteiso*-branched forms, according to compositional analysis (Table 1). Minor peak at *m/z* 1435.6 was matched with a *mono*-phosphorylated tetra-acylated lipid A species carrying two 17:0(3-OH) and two 15:0(3-OH). These observed ions were also accompanied with corresponding sodiated and di-sodiated forms in MALDI-TOF MS spectra recorded in the positive-ion polarity. The positive-ion spectrum of **LipA**_**me**_ (Fig. 5), as an example, displayed two main clusters of peaks that were detected at about *m/z* 1602.5 and *m/z* 1705.5 and assigned as [M-*P* + Na^+^]^+^ and [M + 2Na^+^]^+^ lipid A species, respectively (Table 2). Also in these spectra, minor lipid A species carrying a 2-aminoethyl phosphate (*P*EtN) group was matched with the peak at *m/z* 1762.5 (Fig. 5).

**Table 2:** The main ion peaks observed in the MALDI-TOF MS spectra reported in Figs. 4 and 5, the predicted masses, and the proposed interpretation of the substituting fatty acids, phosphate and *P*EtN, on the lipid A backbone. The observed masses reported in the table are compared to the calculated monoisotopic mass (predicted mass, Da) of each ion based on the proposed lipid A structures. ^a^Ion detected in the negative-ion MALDI-TOF MS spectrum recorded on bacterial cells (Fig. 5)

***Negative-ion polarity***			
**Predicted mass (Da)**	**Observed ion peaks (*m*/*z*)**	**Acyl substitution**	**Proposed composition**
1718.2	1716.7	Penta-acyl	HexN^2^,PEttN,[17:0(3-OH)]^2^ [15:0(3-OH)] [16:0(3-OH)] (15:0)
1674.2	1673.7	Penta-acyl	HexN^2^,P, [17:0(3-OH)]^2^ [15:0(3-OH)] [16:0(3-OH)] (15:0)
1660.2	1659.7	Penta-acyl	HexN^2^,P, [17:0(3-OH)]^2^ [15:0(3-OH)]^2^ (15:0)
1646.2	1645.7	Penta-acyl	HexN^2^,P, [17:0(3-OH)]^2^ [15:0(3-OH)]^2^ (14:0)
1436.0	1435.6	Tetra-acyl	HexN^2^,P, [17:0(3-OH)]^2^ [15:0(3-OH)]^2^
1450.0	1449.6	Tetra-acyl	HexN^2^,P, [17:0(3-OH)]^2^ [15:0(3-OH)] [16:0(3-OH)]
1419.0	1418.2^a^	Tetra-acyl	HexN, HexN3N, P, [17:0(3-OH)]^2^ [15:0(3-OH)] (15:0)
***Positive-ion polarity***			
1764.3	1763.5	Penta-acyl	HexN^2^,PEtN, [17:0(3-OH)]^2^ [15:0(3-OH)] [16:0(3-OH)] (15:0), 2 Na^+^
1720.2	1720.5	Penta-acyl	HexN^2^,P, [17:0(3-OH)]^2^ [15:0(3-OH)] [16:0(3-OH)] (15:0), 2 Na^+^
1706.2	1705.4	Penta-acyl	HexN^2^,P, [17:0(3-OH)]^2^ [15:0(3-OH)]^2^ (15:0), 2 Na^+^
1692.2	1691.4	Penta-acyl	HexN^2^,P, [17:0(3-OH)]^2^ [15:0(3-OH)]^2^ (14:0), 2 Na^+^
1683.2	1682.4	Penta-acyl	HexN^2^,P, [17:0(3-OH)]^2^ [15:0(3-OH)]^2^ (15:0), Na^+^
1669.2	1668.4	Penta-acyl	HexN^2^,P, [17:0(3-OH)]^2^ [15:0(3-OH)]^2^ (14:0), Na^+^
1603.2	1602.5	Penta-acyl	HexN^2^, [17:0(3-OH)]^2^ [15:0(3-OH)] [16:0(3-OH)] (15:0), Na^+^

**Fig. 6:**
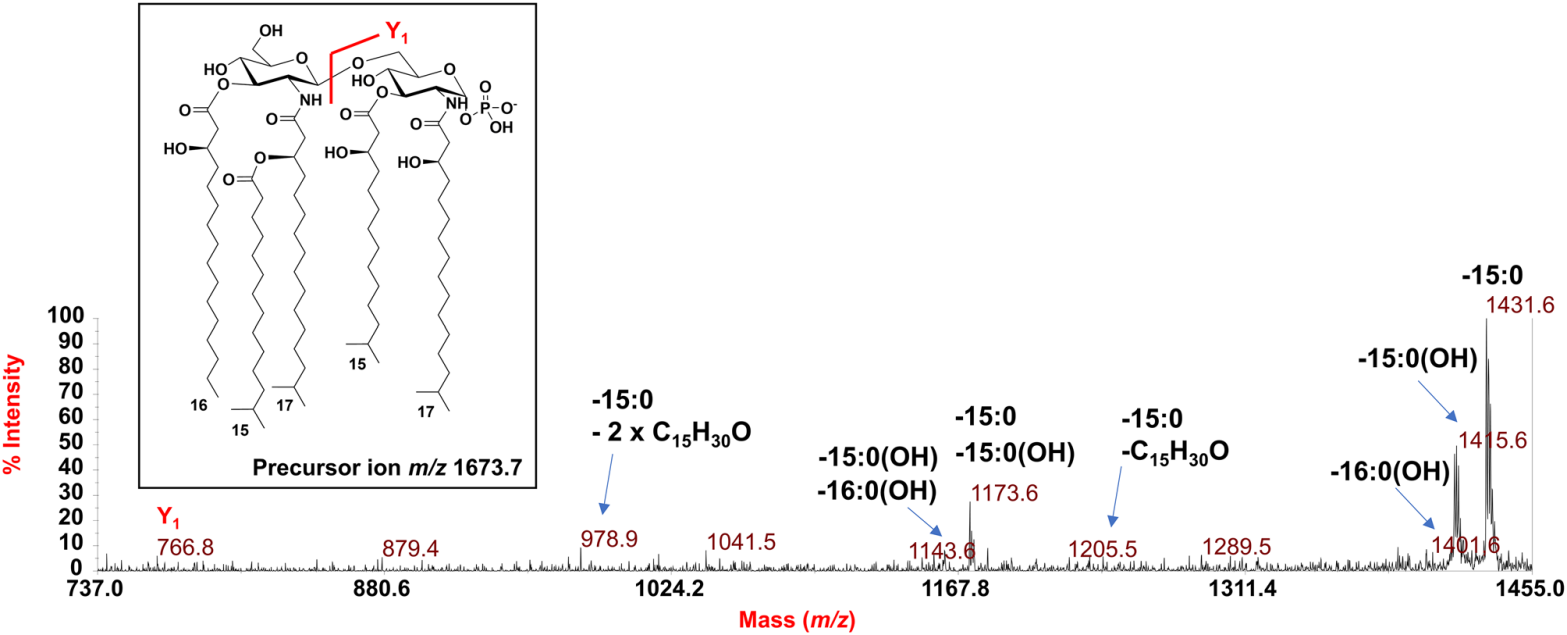
Negative-ion MALDI MS/MS spectrum of precursor ion at *m/z* 1673.7, a representative ion peak of the cluster ascribed to *mono*-phosphorylated penta-acylated lipid A species detected for *Z. profunda* SM-A8*7* **LipA**_**me**_. The assignment of main fragments is shown in the spectrum alongside with the proposed structure that is sketched in the inset. The nature of branched acyl chains (*iso*) in the depicted lipid A structure is given for description purposes only. Peaks derived by the loss of C_15_H_30_O (226 mass units), as a consequence of the rearrangement occurring on *N*-linked 3-OH acyl chains having the hydroxyl group free, have been also indicated

**Fig. 7:**
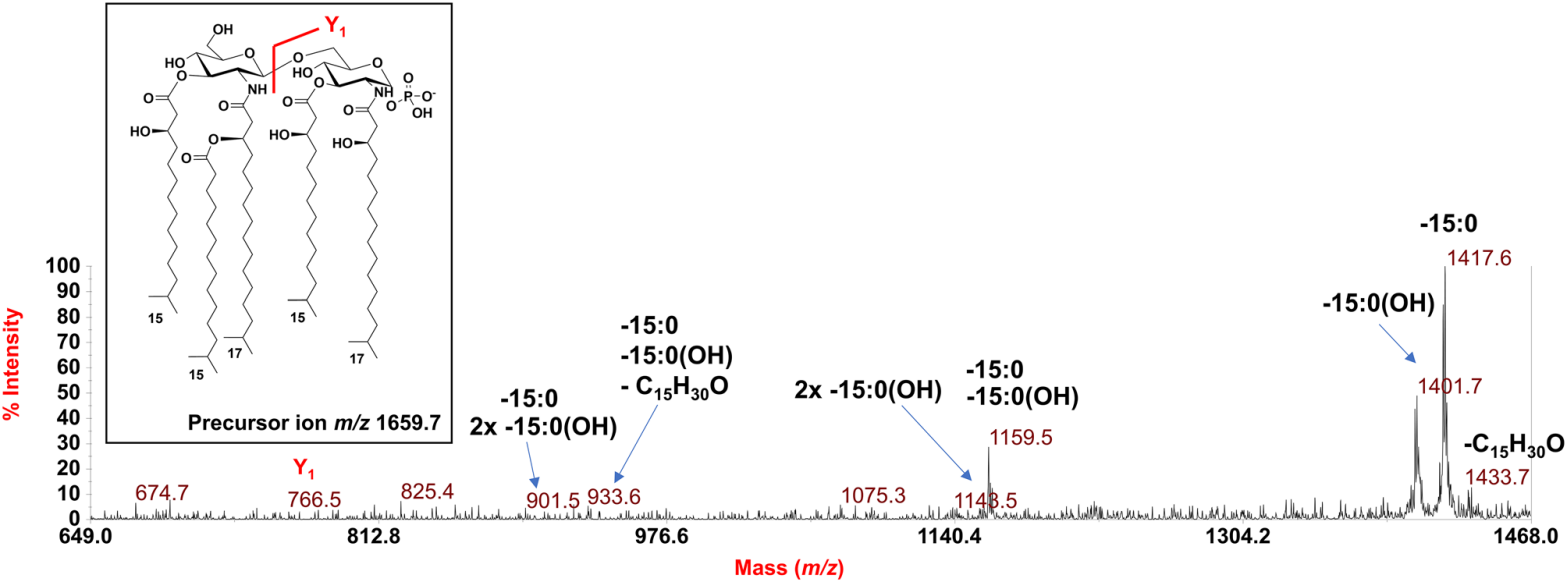
Negative-ion MALDI MS/MS spectrum of precursor ion at *m/z* 1659.7, another representative ion peak of the cluster that matched with *mono*-phosphorylated penta-acylated lipid A species and found for *Z. profunda* SM-A8*7* **LipA**_**me**_. The assignment of main fragments is reported. In the inset it is reported the proposed structure, where the *iso*-branching of the fatty acids is tentative. Peaks originated from the loss of C_15_H_30_O (226 mass units) have been also indicated

To determine the location of the phosphate group and the acyl moieties with the respect to the disaccharide backbone of *Z. profunda* SM-A87 lipid A, a negative-ion MALDI-TOF MS/MS investigation was conducted on several peaks. The MS/MS spectrum of the precursor ion at *m/z* 1673.7 detected in the MALDI-TOF spectrum recorded for **LipA**_**me**_ is reported in Fig. 6. It showed two intense peaks at *m/z* 1431.6 and *m/z* 1415.6 assigned to ions derived from the loss of a 15:0 and a 15:0(3-OH) acyl chain, respectively. A less intense peak was detected at *m/z* 1401.6 and was attributed to a fragment that originated from the loss of a 16:0(3-OH). Peaks at *m/z* 1143.6 and *m/z* 1173.6 were assigned to ions generated by the sequential loss of a 15:0(3-OH) and a 16:0(3-OH) (for the peak at *m/z* 1143.6), and of a 15:0(3-OH) and a 15:0 (for the peak at *m/z* 1173.6). The absence of any peak assignable to an ion originated from the loss of a whole unit of a hydroxylated fatty acid bearing 15:0 as a secondary acyl moiety, suggested that the latter was in an acyloxyacyl amide moiety. This hypothesis was further supported by the detection of the peak at *m/z* 978.9 that was assigned to a fragment generated from a rearrangement occurring only when the *N*-linked fatty acids at positions C-2 and C-2’ have a free 3-OH group, i.e., they do not bear secondary acyl substituents [23]. In this case, in fact, an enamine to imine tautomerization followed by a six-membered ring-based rearrangement was responsible for the loss of a C_15_H_30_O neutral fragment (226 amu) from each primary *N*-linked 17:0(3-OH) thus giving rise to an ion matchable with the peak at *m/z* 978.9 (Fig. 6). Since such a rearrangement was observed only in concomitance with the loss of the 15:0 moiety, it was possible to locate this fatty acid as a secondary substitution of a primary *N*-linked 17:0(3-OH). A less intense peak at *m/z* 1205.5 was matched with a similar ion generated by the loss of the 15:0 moiety plus 226 amu, thus indicating that the above rearrangement only occurred on one primary *N*-linked fatty acid. Finally, the observation of the Y_1_ ion [24] at *m/z* 766.8,which is originated from the cleavage of the glycosidic linkage of the disaccharide backbone, allowed to locate a 17:0(3-OH) and a 15:0(3-OH) on the reducing glucosamine unit, which, in turn, also confirmed the presence of a 17:0(3-OH), a 15:0(3-OH), and the secondary 15:0 acyl moiety on the non-reducing glucosamine. By a similar approach, the precursor ion at *m/z* 1659.7 was also analyzed by negative-ion MS/MS (Fig. 7). The observation of the intense peaks at *m/z* 1401.7 and *m/z* 1417.6, which were matched with ions derived by the loss of a 15:0(3-OH) and a 15:0 respectively, and the detection of peaks at *m/z* 1143.5 and *m/z* 1159.5 originated by the sequential loss of a 15:0 and a 15:0(3-OH) (*m/z* 1159.5), and of two 15:0(3-OH), allowed the identification of the acyl moieties composing this penta-acylated lipid A species. To point the position of these fatty acids and of the phosphate group, once again it was crucial **i)** the observation of the Y_1_ ion at *m/z* 766.5, which confirmed the presence of one 17:0(3-OH) and one 15:0(3-OH) on the reducing glucosamine, and **ii)** the detection of the peak at *m/z* 933.6, which originated from the sequential loss of a 15:0(3-OH) and of the secondary 15:0 moiety, plus 226 amu, due to the enamine to imine tautomerization occurring on one free primary *N*-linked 17:0(3-OH) (Fig. 7).

**Fig. 8:**
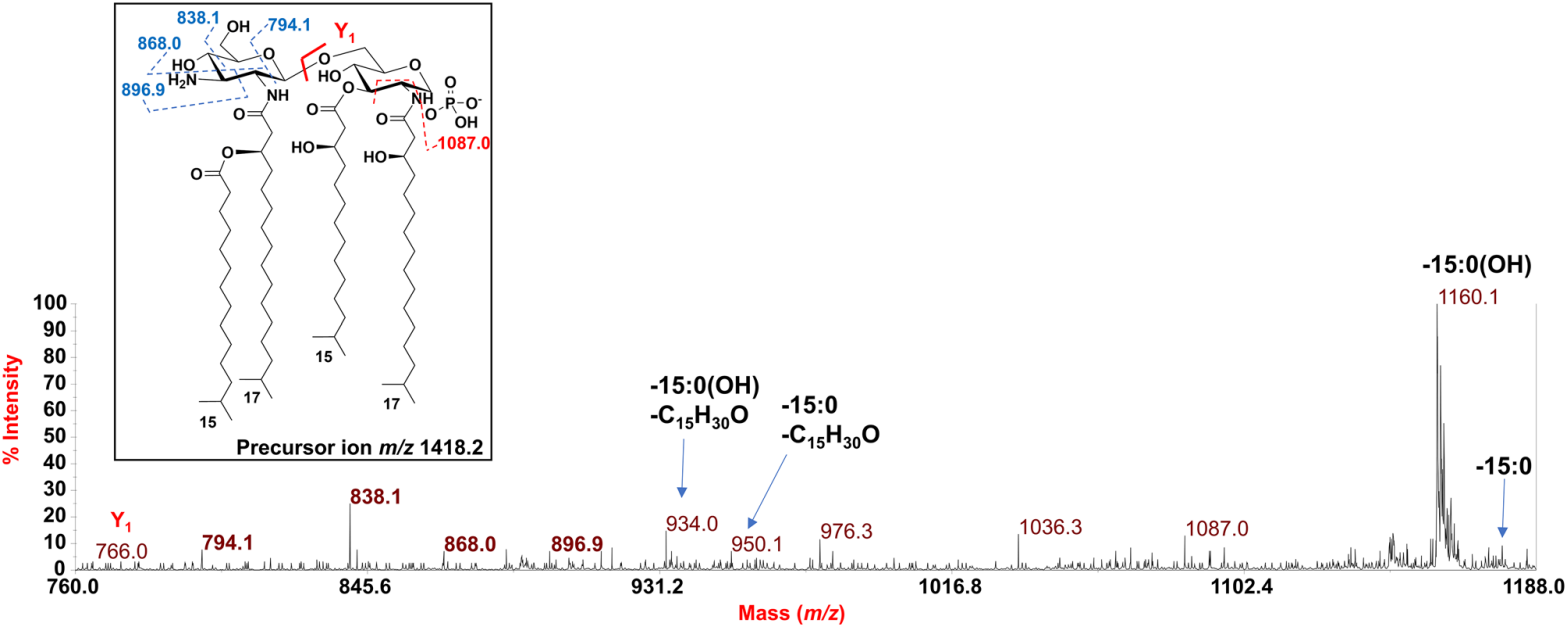
Negative-ion MALDI MS/MS spectrum of precursor ion at *m/z* 1418.2, chosen as representative ion peak of the cluster ascribed to *mono*-phosphorylated tetra-acylated lipid A species and identified by analyzing directly UCF_prec_. In the inset it is reported the proposed structure, where the *iso*-branching of the fatty acids is tentative. Blue dotted lines indicate the cross-ring fragmentations occurred on the non-reducing GlcN3N whose related ion peaks have been reported in bold. Peaks originated from the loss of C_15_H_30_O (226 mass units) have been also reported

To gain more information on the structural heterogeneity of *Z. profunda* SM-A87 lipid A, we took advantage of the MALDI-TOF MS analysis executed directly on bacterial cells (UCF_prec_) to conduct a negative-ion MS/MS investigation on several peaks related to the tetra-acylated lipid A species. As an example, in Fig. 8 is reported the MS/MS spectrum recorded on precursor ion at *m/z* 1418.2. This analysis allowed the identification of a lipid A species composed of the hybrid GlcN3N-GlcN backbone. Briefly, the spectrum clearly showed an intense peak at *m/z* 1160.1 that was matched with an ion generated by the loss of a 15:0(3-OH). In relation to this, the peak at *m/z* 934.0 was identified as belonging to an ion originated from the loss of a 15:0(3-OH) plus 226 amu, i.e. the loss of the neutral C_15_H_30_O fragment described above. Minor peaks at *m/z* 1176.1 and *m/z* 950.1 were assigned to ions resulted from the loss of a 15:0, and of a 15:0 plus 226 amu. The observation of the Y_1_ ion at *m/z* 766.0 pointed that still was a reducing GlcN to carry the phosphate, a 15:0(3-OH) and a 17:0(3-OH). On the other hand, we were able to identify several peaks that were attributed to ions derived by the cross-ring fragmentations (mainly) occurring on the non-reducing sugar unit, which allowed its identification as a GlcN3N (inset of Fig. 8). Such a hybrid backbone, however, was identified only for some minor species, which was also in accordance with compositional analyses.

In conclusion, based on a combination of data from chemical and MS and MS/MS-based structural studies, we propose that *Z. profunda* SM-A87 produces a heterogeneous blend of tetra- and penta-acylated lipid A species, made up of the typical glucosamine disaccharide backbone and, in minor amount, of the hybrid GlcN3N-GlcN backbone, decorated by one phosphate or *P*EtN on the reducing glucosamine unit.

## Discussion

*Z. profunda* SM-A87 is a deep-sea Gram-negative bacterium known to produce high quantities of CPS as an instrument to survive in the adverse conditions of the sea environment. In this study, by handling the cold ethanol precipitation product (CPS-EtOH) previously obtained to isolate the CPS material, we attempted and succeeded in isolating the lipid A fraction of the R-LPS synthesized by this bacterium. Our methodological approach (Scheme 1) entailed both a mild acid hydrolysis of the CPS-EtOH (**LipA**_**ah**_) and an ultracentrifugation of the CPS-EtOH followed by the lipid A (micro)extraction (**LipA**_**me**_) of the so-obtained pellet (UCF_prec_). The employment of chemical and MALDI-TOF MS-based analyses of **LipA**_**ah**_ and **LipA**_**me**_ revealed that the lipid A fraction of *Z. profunda* SM-A87 R-LPS was successfully isolated and showed that it consisted of a heterogenous mixture of species encompassing both tetra- and penta-acylated lipid A forms. This structural deduction was then definitively corroborated by MALDI-TOF MS and tandem MS analysis conducted on bacterial cells (UCF_prec_). The structural complexity of *Z. profunda* SM-A87 lipid A was clearly apparent due to heterogeneity in both the phosphorylation and acylation pattern. As for the phosphorylation pattern, lipid A species carrying one phosphate or one *P*EtN group on the reducing glucosamine units were identified. In addition, *Z. profunda* SM-A87 produces lipid A species that differ not only in the acylation degree (namely tetra- or penta-acylated) but also in the nature and length of the acyl moieties. Worthy of emphasis is certainly the observation of the high content of odd numbered and branched acyl chains, which is a characteristic found also in other bacteria of the Bacteroidetes phylum [25–28], and in the family Flavobacteriaceae [29, 30], of which *Z. profunda* is a member. In particular, saturated, unsaturated, and hydroxylated acyl chains containing 15 and 17 carbon atoms, mostly present as branched structures, were identified as the main fatty acids decorating this lipid A. Strikingly, *Z. profunda* SM-A87 lipid A was found to be very similar to the lipid A isolated from *Flavobacterium meningosepticum* [30] and *Capnocytophaga canimorsus* [29], both belonging to the formerly *Cytophaga*–*Flavobacterium*–*Bacteroides* (CFB) group, which also includes a number of marine bacteria [31–33]. In particular, *Z. profunda* SM-A87 lipid A was analogous to the *C. canimorsus* lipid A that is penta-acylated, lacking the 4′ phosphate and harboring a *P*EtN at the anomeric position of the reducing GlcN of a predominant hybrid GlcN3N-GlcN backbone [29]. However, unlike *C. canimorsus* and *F. meningosepticum*, which are Mammal-associated bacteria, *Z. profunda* SM-A87 decorates its lipid A also with unsaturated acyl chains. This is a well-known characteristic of marine bacteria that tend to increase the desaturation level of their membrane lipids to strategically increment the outer membrane fluidity through decreasing the lipid packing. Cell membrane fluidity is, in fact, a vital *pre*-*requisite* to face with the high hydrostatic pressures of deep-sea environments as a fluid membrane in harsh conditions is essential to maintain the function of important metabolic systems, such as the electron transport chain [34]. Likewise, the observed tendency of expressing branched fatty acids might be related to the enhancement of membrane fluidity and to the lowering of phase transition temperature of the lipid bilayer as a supplement or an alternative to the synthesis of unsaturated acyl chains [35]. Of note, *Z. profunda* SM-A87 showed to largely prefer *anteiso*-branched to *iso*-branched or straight fatty acids for its lipid A. This might be explained by the peculiarity of *anteiso*-positioned methyl groups of causing a greater disturbance of the packing order of the lipid chains, thus resulting in higher fluidity of the membrane [34, 35].

Of note, in addition to heterogeneity in the nature and number of acyl chains as well as in the substitution of the polar head groups of this lipid A, heterogeneity also exists in the amino sugar composition. Indeed, as stated above, we have observed that the hydrophilic backbone of *Z. profunda* SM-A87 lipid A consists of two *mono*-phosphorylated hexosamine disaccharides. In addition to a dominant GlcN-GlcN backbone, a hybrid disaccharide backbone of GlcN3N and GlcN was also identified, although only for minor species. Such an unusual GlcN3N containing backbone is not unprecedented as it has been found to be a component of the lipid A in a number of environmental and plant-associated bacteria, including *Rhodopseudomonas viridis* [36], *R. sulfoviridis* [37], *R. palustris* [23, 36], *Oligotropha carboxidovorans* [38], and *Bradyrhizobium* [39–41]. However, lipid A structures of these bacteria have unique backbones consisting of the diamino monosaccharide only. By contrast, only a few bacteria have been found to express the hybrid backbone and among these the above-mentioned *C. canimorsus* [29] and *F. meningosepticum* [30].

It is worth to note that the lipid A structural characteristics discussed for *Z. profunda* SM-A87, especially the occurrence of unsaturated and branched acyl chains, are certainly of great interest from an evolutionary point of view, advancing knowledge of the molecular tools that may favour deep-sea bacterial adaptation phenomena. However, the discovery of such unusual structural features is even more intriguing under biological and immunological points of view given that the TLR4/MD-2-mediated immunoactivity of an LPS almost entirely relies on the lipid A structure. In this regard, it is well established that lipid A species expressing less than six acyl chains (commonly defined as hypo-acylated lipid As) usually only poorly activate the human TLR4/MD-2-mediated immune response [10–13]. Here we have shown that *Z. profunda* SM-A87 expresses tetra- and penta-acylated lipid A and only one phosphate at position 1 of the reducing glucosamine, which also has been associated to a 100-fold decrease in the immunostimulatory activity of an LPS [42]. Contextually, previous immunological studies on *C. canimorsus* [29] LPS showed that it exerts a less immunostimulant activity than the highly immunogenic hexa-acylated *Escherichia coli* LPS. Of note, the lipid A alone of *C. canimorsus* was shown to be almost not pro-inflammatory [42, 43] at all, thus suggesting that the low immunoactivation observed was dependent on the peculiar structure of the carbohydrate part of this LPS [44]. Given these premises, the immunological properties of *Z. profunda* SM-A87 lipid A are expected to be similar to those observed for *C. canimorsus.* Nevertheless, the occurrence of unsaturated acyl chains, not present in *C. canimorsus* lipid A as well as the higher heterogeneity and tendency to express branched fatty acids, beg the question of whether these structural features impact on the immunological activity of *Z. profunda* SM-A87 lipid A. To this aim, studies are currently in progress in our laboratories.

## Conclusions

In this study, we defined the lipid A structure of *Z. profunda* SM-A87, a Gram-negative bacterium isolated from a deep-sea sediment in the southern Okinawa Trough [5]. We obtained structural information of the lipid A by manipulating the cold ethanol precipitation product (CPS-EtOH, Scheme 1) generated to isolate the CPS of *Z. profunda* SM-A87 and that included both the polysaccharide fraction and bacterial cells. Indeed, once cells were obtained by means of an ultracentrifugation step of CPS-EtOH, a direct MALDI-TOF MS and MS/MS investigations were executed and compared to MS analyses performed on the (micro)-extracted lipid A (**LipA**_**me**_). Likewise, MALDI-TOF MS and MS/MS analyses were conducted on the precipitate formed upon mild acid treatment (**LipA**_**ah**_) of CPS-EtOH, which also was compared to the above MS studies (Scheme 1). All the analyses confirmed the isolation of the lipid A fraction and converged on a heterogenous mixture of tetra- and penta-acylated lipid A species carrying one phosphate or one *P*EtN on the reducing glucosamine unit. A high content of branched, odd-numbered as well as unsaturated fatty acids were the peculiar structural features of this lipid A, whose hydrophilic backbone was found to be also composed, in minor amount, of the hybrid disaccharide GlcN3N-GlcN.

## Data Availability

The datasets used and/or analyzed during the current study are available from the corresponding author on reasonable request.

## Abbreviations

CPS: Capsular polysaccharide
CPS-EtOH: Cold ethanol precipitation product
DHB: 2,5-Dihydroxybenzoic acid
EI: Electron impact
GC-MS: Gas Chromatography-Mass Spectrometry
GlcN: 2-Amino-2-deoxy-D-glucose
GlcN3N: 2,3-Diamino-2,3-dideoxy-D-glucose
LipA_ah_: Lipid A fraction obtained by means of mild acid hydrolysis of CPS-EtOH
LipA_me_: Lipid A fraction obtained by applying the lipid A micro-extraction protocol
LPS: Lipopolysaccharide
MALDI: Matrix-Assisted Laser Desorption Ionization
MAMP: Microbe Associated Molecular Pattern
MD-2: Myeloid Differentiation factor-2
MS: Mass Spectrometry
OS: Oligosaccharide
*P*EtN: 2-Aminoethyl phosphate
PRR: Pattern Recognition Receptor
R-LPS: Rough-type Lipopolysaccharide
SDS-PAGE: Sodium Dodecyl Sulfate–Polyacrylamide Gel Electrophoresis
S-LPS: Smooth-type Lipopolysaccharide
THAP: 2′,4′,6′-Trihydroxyacetophenone
TLR4: Toll-Like Receptor 4
TOF: Time Of Flight
UCF_prec_: Ultracentrifugation precipitation product
